# Medical News and Miscellany

**Published:** 1893-03

**Authors:** 


### Medical News and Miscellany.

The Texas Legislature, now in session, has passed an act making it a penal offense in a physician to give a prescription for whisky without an examination. It is intended to meet a too prevalent practice of prescribing whisky for people on Sunday when the saloons are closed.

Dr. J. B. S. Holmes' splendid Infirmary at Rome, Ga., was destroyed by fire on 26th January last, together with all its contents. The Doctor advises us that it will be immediately rebuilt and equipped, and put upon an even more splendid footing than before. When completed it will be duly announced.

We learn that a bill to create a State Board of Health was gotten up in Galveston, by whom we did not ascertain, and the

Galveston Senator was requested to introduce it. *If introduced* it will die in the committee room, as have all those which have gone before. The present health system seems good enough; it is economical and efficient.

---

**The Quarantine** steamer "Bessie Ross," which for two years has been lying unused at Harrisburg and which the Twenty-second Legislature by a special act authorized the State Health Officer to sell, has just been sold for $3000 to Captain J. Ward, of Houston. Last year she was advertised in Galveston and Houston for sale to the highest bidder, and $3000 was the best bid received.

---

**At time** of going to press the bill regulating the practice of medicine (published in full in our January number), which was gotten up by Dr. Wooten, and the main feature of which was to require a diploma from a three years' school, has not emerged from the committee room in the Legislature, the grave of innumerable predecessors. The bill pleased the homeopaths immensely, because it requires no examination, and recognizes the present status of those who have complied with the law.

---

**Massage Treatment.**—Massage is recognized by the regular profession as a legitimate feature of therapeutics. There has been in Austin, San Antonio, Dallas, and other Texas cities, recently, a lady who is said to be very skillful in this health-giving art, Madame Tully, and she brought with her proper credentials from leading physicians. She also had testimonials from prominent Texas physicians to the effect that she had successfully treated certain patients of theirs. She cures rheumatism, sciatica, and nervous diseases, and develops the muscular system. Madam Tully also treats the face, and cures pimples, black-heads and blotches. She will make a tour of Texas, and finally locate in one of our larger cities. Address her at 844 Main street, Buffalo, N. Y.

---

**In Illinois,** since Pfifer was snowed under, the governor who so loved homeopaths as to appoint a hemeopathic kid surgeon general, and a Democratic governor has been elected, it is said there will be a clean sweep of Republican office holders. The medical profession of Illinois were so indignant against Pfifer that they *organized* and voted solidly against him. *Now* there is a demand

—according to Illinois papers—for competent Democratic physicians to fill the various medical offices; and it said, as the law requires "experience" at the hands of the appointee, there is but one Democratic physician in the State eligible to be Superintendent of the State Asylum, and he will not have it. It pays $3500 a year and found. Illinois is looking to Texas for competent Democratic doctors.

---

Dr. H. C. Ghent's daughter, Laura, was married in Belton on 16th inst., to Dr. M. S. Graves, of Waco.

---

The Health Restorative Company have paid that advertising bill we offered for sale last month—just four months after it was due.

---

Quarantine Conference.—At the time of going to press (16th) there is in session in Washington a conference of State Quarantine Officers, called by Supervising Surgeon-General Waller Wyman, of the Marine Hospital Service. Section five, of the National Quarantine Law recently enacted by Congress, requires that rules and regulations to govern ships at starting, during voyage and arrival at American ports, shall be made; and Surgeon-General Wyman has asked the Secretary of the Treasury, who is the nominal head of the Quarantine Department ! ! that Ex-Surgeon-General John B. Hamilton, Surgeon Giddings and some other be appointed *a board* to get up said rules. Of course the request was granted, and this board convened in Washington on 18th February, ult. Just what this conference is for, therefore, is conjectural; supposed to be for the purpose of agreeing upon some uniform system for coast quarantine.

Our State Health Officer, R. M. Swearingen, left Austin on the 12th to attend the conference, on a special invitation, Mrs. Swearingen accompanying him.

---

At a dinner given by the Galveston Medical Club, not long ago, and after the fourth course of wine had commenced to flow freely, impromptu speeches were called for. Dr. X., when called on for his experience, asked to be permitted to render it in poetry [?], which, being granted, he arose and said:

"Mr President and gentlemen, allow me to give an example of only one of my

### HALCYON REMINISCENCES.

"*Pictoribus atque poetas, quidlibet audendi fuit semper asque potestescas,*" and so forth.

I arise to a question of justice,
To a privileged question I rise,
For I wish to relate here, this evening,
A case that was "covered with flies."

Not actually covered with insects,
But fig'ratively clad, so to say,
For alluding to flies on a person
Is one of the "slangs" of the day.

'Twas a maiden whose case I encountered,
A maiden though fair was not frail,
A maiden who cared not for fortune,
So the drug stores in town did not fail.

'Twas a maiden of strange fascination,
A lady of wonderful ways,
An old maid, without hesitation,
And they called her Octavia Hayes.

Now Octavia Hayes was a daisy,
But I always thought her head level,
While many considered her crazy,
And some even called her the devil.

She has blossomed some forty odd summers,
And, if everything's true that was said
By doctors and ladies and preachers,
She had spent about thirty in bed.

Her weakest point seemed to be "misrey,"
But misery not well defined,
For sometimes 'twas over the pubis
And sometimes 'was seated behind.

A number of Galveston doctors
Had taken a peep at her os,
And had penciled it over with silver,
And had stuffed her with cotton and moss.

Now Octavia Hays,
With her singular ways,

Knew all the best doctors of Texas;
  They had seen her face,
And had "pulled at her case,"
  Ere she came to our city to vex us.

They had looked at her organs
  From ankles to nose;
They had treated her down
  From her hair to her toes,
And what they had left out,
  Why — — — nobody knows.

She came to our city
  In anguish and want;
Was wasted in finance,
  Her anatomy gaunt;
She went for our doctors,
  And ladies, so kind;
She asked for the best
  That our people could find.

And unless it came quickly
  She raised merry Kain,
Till at last our good ladies
  Were kept on a strain
To supply her demands
  And Miss H. entertain.

  A custom she had,
  Which you all might call bad,
Was to send for a doctor in a hurry,
  And if you came late,
  You had better talk straight,
Or else there would be a small flurry,
  For her tongue it was long,
  And her voice it was strong,
And a giant would have called her a worry.

Another queer custom had Octavia Hays;
'T was to call at my office, on beautiful days,
  And from noon-time till dark
  She would sit there and talk
Of all the old ailments I had heard o'er and o'er;
It caused me to keep a strict watch on my door.

Our doctors did all in their power they could,
To allay her condition and do her some good;
We filled her with medicine and ordered her food.

We blistered her back; at her rectum we whacked,
'Thout finding the lesion that ailed her;
We rubbed down her spine till the skin it did shine,
And all our kind service but failed her.

With pill and with powder we often did crowd her,
But she criod the louder: "Give something more strong!"
We doubled our blisters, gave capsicum clysters,
'Twas alwas this: "Misters, you're all in the wrong;

"Can't you use your big spectrum and look at my rectum?
You can cure me if ever you try;
Or give an injection after my own direction,
If you don't I will certainly die?"

Well, nothing would please her, we all seemed to tease her,
And medical men of sober mien
Were oft seen to giggle and sometimes to wriggle,
While madam gave orders or vented her spleen.

At last the profession, with one loud confession,
Declared her a nuisance aud closed on her case;
When sent for to treat her, they said they would meet her,
Oh yes, they would greet her—in some other place.
But my poem I care not, right here, to disgrace,
By giving the name of that place any space;
Suffice it to say, though I loathe it to tell,
It was Hades or Houston—but oftener 'was——well.

Alas, she has landed, and I fear she has stranded
In a haven of pleasure, "dot rot her."
But she's contented, and the profession's resented;
THE MEDICAL STUDENTS HAVE GOT HER!

